# The evolving landscape of regulatory T cells in leukemia: from mechanisms to advanced immunotherapeutic strategies

**DOI:** 10.3389/fimmu.2026.1822997

**Published:** 2026-05-20

**Authors:** Huili Xu, Xiaoli Zhang, Jun Zhang

**Affiliations:** Department of Hematology, Pingdingshan First People’s Hospital, Pingdingshan, Henan, China

**Keywords:** chimeric antigen receptor T cells, immune evasion, immunotherapy, leukemia, regulatory T cells, tumor microenvironment

## Abstract

The tumor microenvironment (TME) in hematologic malignancies constitutes a dynamic ecosystem supporting leukemogenesis, therapeutic resistance, and immune evasion. Key drivers of this immunosuppressive network are regulatory T cells (Tregs), a CD4+ subset conventionally defined by FoxP3 and CD25 expression. While physiological Tregs are essential for self-tolerance, leukemic blasts frequently co-opt these cells to dampen anti-tumor immunity. Here, we review the distinct roles of Tregs across the spectrum of leukemias, including Acute Myeloid Leukemia (AML), Acute Lymphoblastic Leukemia (ALL), Chronic Myeloid Leukemia (CML), and Chronic Lymphocytic Leukemia (CLL). We dissect the molecular machinery governing Treg recruitment and suppression within the bone marrow niche, highlighting the CCL22/CCR4 axis, metabolic reprogramming via the CD39/CD73 adenosine pathway, and indoleamine 2,3-dioxygenase (IDO)-mediated tryptophan catabolism. Additionally, we critically evaluate the nuanced and often discordant prognostic impact of Treg infiltration across different subtypes. A significant portion of this review examines the evolving therapeutic landscape, scrutinizing Treg-depleting antibodies (e.g., mogamulizumab, RG6292), immunomodulatory small molecules (venetoclax, hypomethylating agents, TKIs), and the challenges Tregs pose to cellular therapies. Finally, we discuss novel strategies to circumvent Treg-mediated resistance, offering a perspective on restoring anti-leukemic immunity.

## 1 Introduction

Cancer immunosurveillance acts as a fundamental physiological barrier to tumorigenesis, recognizing and eliminating malignant cells. Leukemia development, therefore, represents a breach of this surveillance mechanism, often driven by a profoundly immunosuppressive tumor microenvironment (TME). Among the cellular constituents of the hematopoietic TME, regulatory T cells (Tregs) have emerged as critical mediators of immune tolerance (1). While originally characterized for preventing autoimmunity, in oncology, Tregs shield malignant cells from cytotoxic attack by CD8+ effector T cells and NK cells (2).

In hematologic malignancies, Treg function is context-dependent due to the unique immunological architecture of the bone marrow (BM) and secondary lymphoid organs. Unlike solid tumors, where Tregs infiltrate a discrete mass, leukemic Tregs operate within the hematopoietic niche, engaging in bidirectional crosstalk with leukemic stem cells (LSCs), stromal cells, and developing immune effectors (3). Leukemic cells not only recruit Tregs via chemokine gradients but also actively drive their differentiation through metabolic manipulation and direct cell-contact (1).

The clinical relevance of Tregs is underscored by their association with poor prognosis and treatment failure (4). However, this relationship is complex; in specific contexts, such as early-stage inflammation-driven tumorigenesis or post-transplant settings, Tregs may play paradoxical roles (1, 5). Furthermore, the advent of immunotherapy has highlighted Tregs as a significant barrier to efficacy. For instance, relapse in B-cell ALL patients treated with blinatumomab or CD19 CAR-T cell therapy has been mechanistically linked to the persistence of suppressive Tregs (6).

This review synthesizes preclinical and clinical data to elucidate the biological underpinnings of Treg function in leukemia and evaluate the potential of targeting this population therapeutically. To ensure a comprehensive and rigorous overview of the landscape of regulatory T cells in leukemia, a structured literature search was conducted. We systematically searched PubMed and Web of Science for articles published from their inception up to March 2026. The search strategy utilized the following keywords and Medical Subject Headings (MeSH) terms in various combinations: (“Regulatory T cells” OR “Tregs” OR “FoxP3”) AND (“Leukemia” OR “Acute Myeloid Leukemia” OR “AML” OR “Acute Lymphoblastic Leukemia” OR “ALL” OR “Chronic Lymphocytic Leukemia” OR “CLL” OR “Chronic Myeloid Leukemia” OR “CML”) AND (“tumor microenvironment” OR “immunotherapy” OR “immune evasion” OR “CAR-T cells” OR “BiTEs”). The inclusion criteria for study selection were: (1) peer-reviewed original research articles, clinical trial reports, and comprehensive reviews; (2) studies specifically investigating the phenotypic, prognostic, or therapeutic aspects of Tregs within the context of hematologic malignancies; and (3) articles published in English. Duplicate records, non-peer-reviewed preprints, and studies strictly focusing on solid tumors without relevance to leukemia were excluded. This systematic approach ensures that the synthesized data reflects both foundational mechanistic insights and the latest clinical advancements in Treg-directed therapies.

## 2 Biology and suppressive mechanisms of Tregs in the leukemic niche

### 2.1 Phenotypic definition and heterogeneity

Tregs are classically defined as CD4+CD25+FoxP3+ T cells. The transcription factor FoxP3 acts as the master regulator of lineage stability, controlling the expression of tolerance-inducing molecules. However, within the inflammatory milieu of leukemia, this phenotypic definition can be ambiguous. Activated non-regulatory effector T cells may transiently upregulate FoxP3 and CD25, necessitating sophisticated immunophenotyping to identify bona fide Tregs (1). Robust identification panels typically include low CD127 expression alongside high expression of inhibitory receptors such as CTLA-4, TIGIT, and ICOS (4).

Tregs in the TME exhibit considerable heterogeneity, comprising thymus-derived natural Tregs (nTregs) and peripherally induced Tregs (pTregs or iTregs). In leukemia, Treg pool expansion results from both nTreg recruitment and the *de novo* conversion of CD4+CD25- naive T cells into FoxP3+ iTregs, a process driven by leukemia-derived cytokines like TGF-β (7). Single-cell RNA sequencing has further delineated this complexity, identifying specific subsets—such as TNFR2+ Tregs—that possess enhanced suppressive capacity and are preferentially enriched in the acute myeloid leukemia (AML) microenvironment (8).

### 2.2 Recruitment and homing dynamics

**Figure 1:**
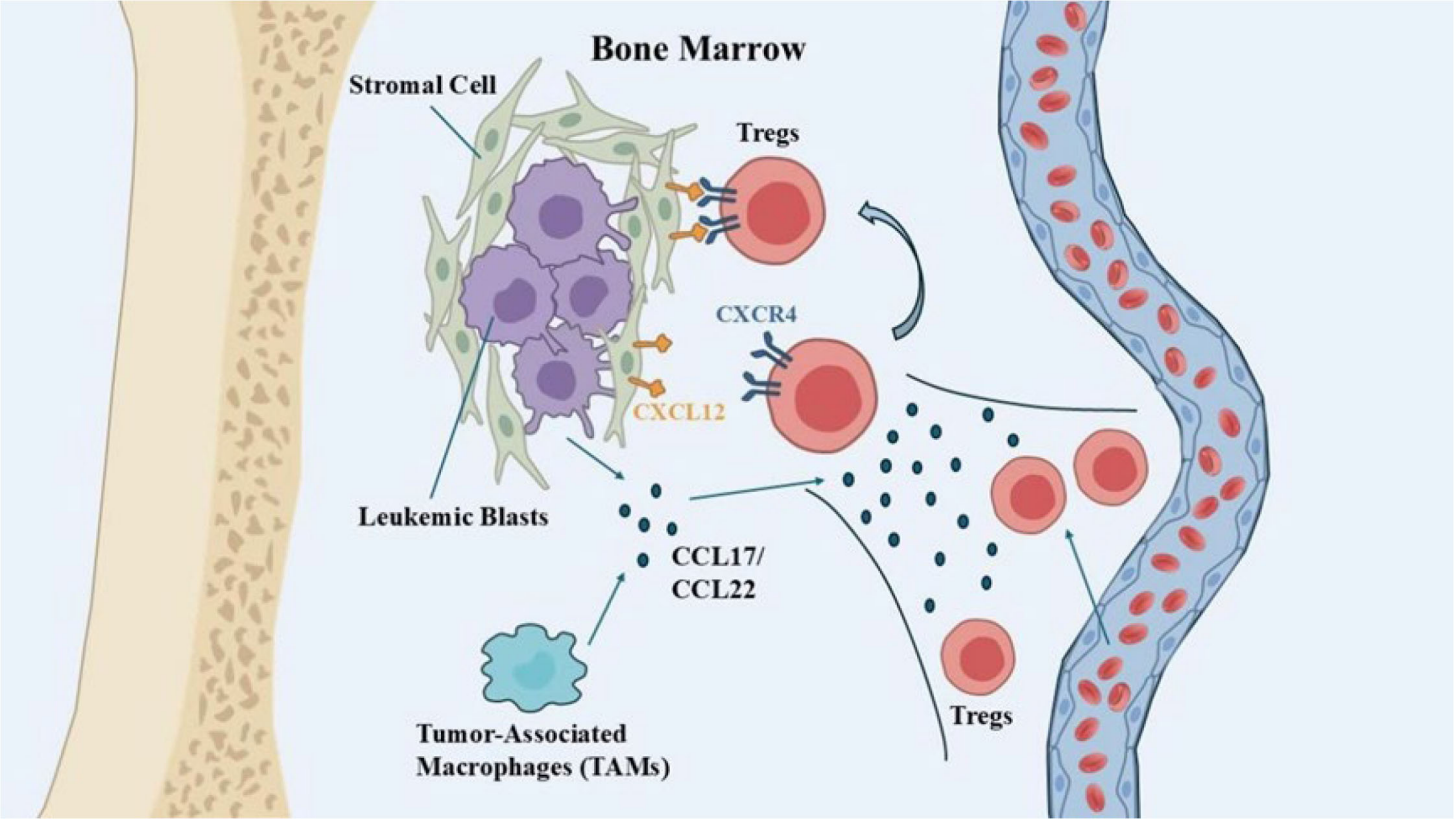
Mechanisms of Treg recruitment in the leukemic microenvironment.

The accumulation of Tregs in the bone marrow and peripheral blood is not stochastic but actively orchestrated by chemokine networks (**Figure 1**).

#### 2.2.1 The CCR4-CCL17/CCL22 axis

A well-characterized mechanism involves the secretion of CCL17 (TARC) and CCL22 (MDC) by leukemic blasts and tumor-associated macrophages (TAMs) (9). Tregs constitutively express the receptor CCR4 and migrate along this gradient into the tumor niche. In adult T-cell leukemia/lymphoma (ATLL) and AML models, high CCL22 levels correlate directly with increased Treg infiltration (9). This axis represents a viable therapeutic target, as evidenced by the efficacy of CCR4 blockade in clinical trials (10).

#### 2.2.2 The CXCR4/CXCL12 axis and bone marrow retention

The bone marrow niche constitutively expresses CXCL12 (SDF-1) to retain hematopoietic stem cells (HSCs) via CXCR4. Tregs also utilize this axis to home to the bone marrow (11). Crucially, this allows Tregs to co-localize with Leukemic Stem Cells (LSCs), creating an immune sanctuary. This spatial proximity is vital for maintaining LSC quiescence and therapeutic resistance (3, 12).

#### 2.2.3 Alternative chemokine networks

Pathways such as the CCR6-CCL20 axis and the CXCR3 ligand system recruit specific Treg subsets that mimic the trafficking patterns of Th1 or Th17 effector cells (9). This “chemokine mirroring” ensures suppressors are positioned in direct proximity to their targets, maximizing immunosuppressive efficiency. .

The accumulation of Tregs in the bone marrow and peripheral blood is not random but actively orchestrated by specific chemokine axes. On one hand, leukemic blasts and tumor-associated macrophages (TAMs) secrete CCL17 and CCL22, which bind to CCR4 on Tregs and guide their migration into the tumor microenvironment. On the other hand, bone marrow stromal cells constitutively express CXCL12, which mediates Treg homing and retention in the bone marrow via CXCR4. This enables Tregs to co-localize closely with leukemic stem cells (LSCs), establishing an immune-privileged sanctuary that maintains LSC quiescence and promotes therapeutic resistance.

### 2.3 Mechanisms of immune suppression

**Figure 2:**
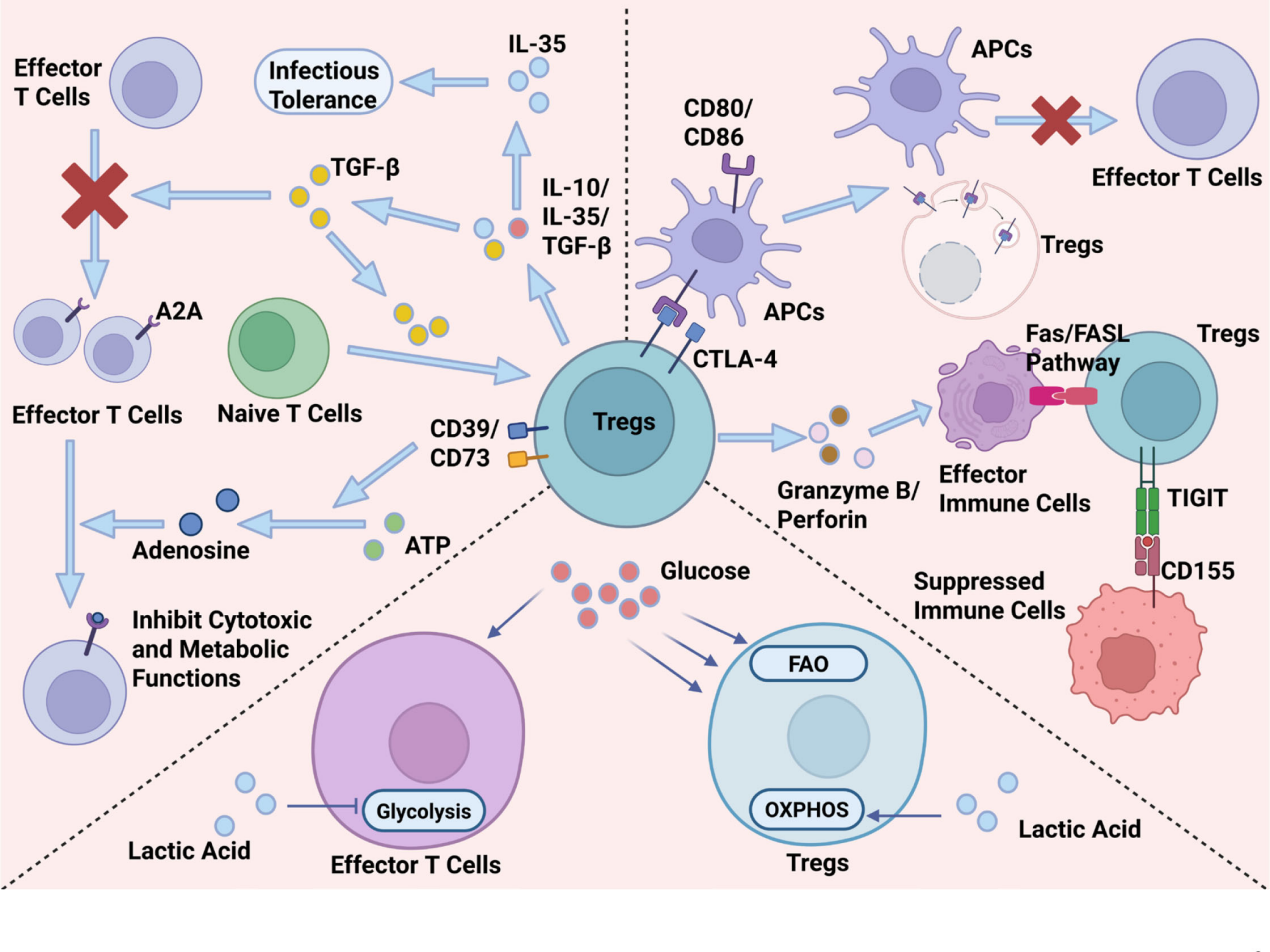
Multidimensional mechanisms of Treg-mediated immune suppression.

Once localized to the leukemic microenvironment, Tregs employ diverse suppressive modalities, broadly categorized into contact-dependent and contact-independent pathways (**Figure 2**).

#### 2.3.1 Soluble mediators: cytokines and metabolites

Tregs secrete high levels of IL-10, TGF-β, and the heterodimeric cytokine IL-35 (13). TGF-β is particularly critical in the leukemic context, as it not only inhibits the proliferation of effector T cells but also drives the conversion of additional naive T cells into Tregs, creating a self-reinforcing loop of immunosuppression (7). IL-35 has been identified as a potent inhibitor that promotes infectious tolerance, spreading the suppressive phenotype to other cells (7, 13).

Leukemic Tregs express high surface levels of the ectonucleotidases CD39 and CD73. These enzymes hydrolyze extracellular ATP (an inflammatory danger signal) into ADP, AMP, and finally adenosine (14). Adenosine binds to A2A receptors on effector T cells, potently inhibiting their cytotoxic and metabolic functions (15). This pathway is highly active in AML and CLL, contributing significantly to the hyporesponsive state of T cells in these patients (16). Notably, high CD39 expression on Tregs correlates with superior inhibitory capacity and resistance to transdifferentiation into Th17 cells (17).

#### 2.3.2 Contact-dependent mechanisms

Tregs constitutively express CTLA-4, which binds CD80/CD86 on antigen-presenting cells (APCs) with high affinity. This interaction strips costimulatory molecules from the APC surface via trans-endocytosis, rendering APCs incapable of activating effector T cells (18).Tregs can directly kill effector immune cells through the release of Granzyme B and Perforin, or by inducing apoptosis via the Fas/FasL pathway (19). Recent studies have highlighted the upregulation of TIGIT on leukemic Tregs. TIGIT competes with the costimulatory receptor CD226 for ligand binding (CD155) and directly transmits inhibitory signals to immunocytes (20). The co-expression of TIGIT and other checkpoints like LAG-3 and PD-1 marks a population of highly suppressive, exhausted Tregs often found in high-risk leukemia (21).

#### 2.3.3 Metabolic competition and reprogramming

Tregs possess a distinct metabolic profile compared to effector T cells. While activated effectors rely on glycolysis, Tregs preferentially utilize oxidative phosphorylation (OXPHOS) and fatty acid oxidation (FAO) (22). In the nutrient-deprived leukemic niche, Tregs outcompete effector cells for glucose. Furthermore, leukemic cells often produce high levels of lactic acid (Warburg effect). While lactic acid inhibits effector T cell function, Tregs are metabolically adapted to thrive in high-lactate environments, utilizing it to fuel suppression (23). This metabolic asymmetry confers a survival advantage to Tregs in the hostile TME. .

(1) Suppression via soluble mediators:

Tregs secrete immunosuppressive cytokines including IL-10, TGF-β, and IL-35. TGF-β not only inhibits effector T cell proliferation but also drives the conversion of naïve T cells into Tregs, creating a self-reinforcing immunosuppressive loop. Additionally, Tregs highly express the ectonucleotidases CD39 and CD73, which hydrolyze extracellular ATP into adenosine. Adenosine binds to A2A receptors on effector T cells, potently suppressing their cytotoxic and metabolic functions.

(2) Contact-dependent suppression:

Tregs constitutively express CTLA-4, which binds with high affinity to CD80/CD86 on antigen-presenting cells (APCs), leading to their removal from the APC surface via trans-endocytosis and thereby disabling T cell co-stimulation. Tregs can also directly kill effector immune cells through Granzyme B/Perforin or induce apoptosis via the Fas/FasL pathway. Moreover, TIGIT is upregulated on leukemic Tregs; it competes with the costimulatory receptor CD226 for binding to CD155 and delivers direct inhibitory signals to immune cells.

(3) Metabolic competition and reprogramming:

Tregs preferentially utilize oxidative phosphorylation (OXPHOS) and fatty acid oxidation (FAO), whereas activated effector T cells rely on glycolysis. In the nutrient-deprived leukemic microenvironment, Tregs outcompete effector T cells for glucose. Furthermore, while lactic acid—produced abundantly by leukemic cells via the Warburg effect—inhibits effector T cell function, Tregs are metabolically adapted to tolerate and even exploit high lactate levels to fuel their suppressive activity. This metabolic asymmetry grants Tregs a survival and functional advantage within the hostile tumor microenvironment.

## 3 Regulatory T cells in Acute Myeloid Leukemia

Acute Myeloid Leukemia (AML) is primarily a disease of the bone marrow (BM), where the leukemic stem cell (LSC) niche plays a pivotal role in disease progression and immune evasion. In the treatment-naive setting, the BM microenvironment is profoundly immunosuppressive. Compared to the peripheral blood (PB), the BM niche is specifically enriched with phenotypically distinct, highly suppressive Tregs (24). The recruitment of these Tregs to the BM is heavily dependent on the CXCR4/CXCL12 and CCL22/CCR4 axes, while their localized expansion is driven by metabolic reprogramming (e.g., IDO expression by blasts) and interactions with bone marrow stromal cells (24).

In AML, the immune landscape is profoundly dysregulated. Studies consistently demonstrate elevated frequencies of Tregs in the PB and BM of treatment-naive AML patients compared to healthy donors (1, 25). These Tregs typically display an activated phenotype (CD45RA-FoxP3hi) and enhanced suppressive potency (26). Treg accumulation is driven directly by the blast population. AML blasts induce FoxP3+ Treg differentiation from CD4+CD25- precursors in the local microenvironment, often mediated by Indoleamine 2,3-dioxygenase (IDO) (27). AML blasts constitutively express IDO, which catabolizes tryptophan into kynurenine. Tryptophan depletion induces effector T cell arrest, while kynurenine metabolites bind the Aryl Hydrocarbon Receptor (AHR) on T cells, skewing differentiation toward a Treg phenotype (27). Clinical studies confirm that IDO expression in AML blasts correlates with higher Treg frequencies and poorer outcomes (28). The kynurenine-AHR axis also promotes the expression of FoxP3, further stabilizing the Treg lineage in the leukemic milieu (29).

The prognostic value of Tregs in AML has been a subject of considerable debate. The majority of studies identify high Treg frequency in the PB or BM at diagnosis as an independent predictor of poor clinical outcomes, including shorter overall survival (OS) and higher relapse rates (30). For instance, a study involving 81 newly diagnosed AML patients found that high expression of LAG-3 and CTLA-4 on T cell subsets, which correlates with Treg burden, was significantly associated with shorter disease-free survival (DFS) (31). Similarly, another study demonstrated that patients with high PD-1+ Treg frequencies had significantly shorter OS and DFS (32).

However, conflicting data exist. Some early reports suggested that in specific contexts, Tregs might be associated with better outcomes, potentially by suppressing inflammation that supports leukemic growth (1). This paradox may be resolved by examining Treg subsets. A critical study identified that the subset of Tregs expressing TNFR2 (Tumor Necrosis Factor Receptor 2) is maximally suppressive. In a murine model and human AML samples, the specific accumulation of TNFR2+ Tregs within the BM was linked to immune evasion, and their depletion restored anti-leukemic immunity (8). Thus, the total Treg number may be less informative than the density of specific, highly suppressive subsets (e.g., TNFR2+, TIGIT+, or ICOS+ Tregs). Recent work characterizing the treatment-naive bone marrow immune microenvironment identified an “inflammation-associated gene score” (iScore) where atypical B cells and CD8+GZMK+ T cells were increased alongside Tregs, correlating with poor survival, further reinforcing the link between a specific inflammatory-suppressive niche and adverse outcomes (33).

Recent insights reveal that Tregs do not distribute randomly but co-localize with Leukemic Stem Cells (LSCs) within the BM niche, creating a zone of immune privilege (3). LSCs exploit Tregs to shield themselves from NK cell and CTL-mediated elimination. Furthermore, Tregs may directly support LSC stemness via soluble factors, although the specific molecular mediators remain under investigation. Notably, Treg depletion in AML models mobilizes LSCs and sensitizes them to chemotherapy, implicating Tregs as a structural component of the LSC niche (3). Interactions via the CXCR4/CXCL12 axis facilitate the retention of both cell types in the vascular niche, promoting mutual survival and quiescence (34).

## 4 Regulatory T cells in Acute Lymphoblastic Leukemia

Unlike AML, Acute Lymphoblastic Leukemia (ALL) encompasses biologically distinct B-cell (B-ALL) and T-cell (T-ALL) lineages with systemic dissemination involving the PB, BM, and frequently the central nervous system and secondary lymphoid organs. The tissue context fundamentally dictates Treg dynamics. While Tregs rapidly expand in the PB and BM of treatment-naive B-ALL patients—driven primarily by blast-derived cytokines—their role and identification in T-ALL are far more complex, as T-ALL blasts themselves can occasionally exhibit a regulatory phenotype or share phenotypic overlaps (such as CD25 expression) with physiological Tregs (35).

In ALL, Treg function exhibits a striking dichotomy based on patient age and disease biology. In pediatric ALL, contrary to the pattern seen in AML, some cohorts indicate that higher bone marrow Treg frequencies at diagnosis correlate with superior outcomes or lack significant adverse prognostic value (36). This discrepancy likely reflects the distinct molecular drivers of pediatric leukemia (e.g., ETV6-RUNX1 fusions) versus adult forms, alongside the fundamentally different immune competence of the pediatric host. Conversely, in adult B-cell precursor ALL (BCP-ALL), Tregs are predominantly deleterious. Elevated Treg levels in treatment-naive and relapsed peripheral blood and bone marrow characterize high-risk disease (6). Mechanistically, this expansion is fueled by lymphoblast-derived TGF-β and the overexpression of adenosine-generating enzymes (37). This age-related divergence underscores that the host immune context is as critical as the leukemic clone in dictating Treg function.

The suppressive barrier imposed by Tregs is a critical determinant of efficacy for T-cell engaging therapies in the relapsed/refractory setting. Blinatumomab, a CD19/CD3 bispecific T-cell engager (BiTE), relies on endogenous T cell fitness. Pioneering work by Duell et al. established that blinatumomab response correlates inversely with peripheral Treg percentage. A discrete cutoff of 8.525% Treg in the PB frequency was identified, capable of predicting 100% of responders and excluding 70% of non-responders (6). While a more recent pooled analysis questioned the universal applicability of this specific cutoff across all trials, the biological principle that high systemic Treg burdens impede BiTE efficacy remains robust (38). Mechanistically, blinatumomab unintentionally activates Tregs in the presence of ALL blasts. These activated Tregs release massive amounts of IL-10, which shuts down the proliferation and cytotoxic activity of the effector T cells that the drug is attempting to engage (6). This suppression is contact-dependent and highly potent, effectively neutralizing the therapeutic mechanism of action. The activated Tregs also express high levels of CD25, acting as an IL-2 sink, further depriving effector T cells of necessary growth factors.

Similar dynamics are observed in CD19 CAR-T therapy for ALL. High burdens of Tregs in the PB and BM can inhibit CAR-T expansion and persistence. A study indicated that higher circulating Tregs, especially one week after infusion, were predictive of shorter relapse-free survival (RFS) and overall survival (OS) (39). Tregs may induce CAR-T exhaustion or limit their access to the BM tumor site. Resistance mechanisms involving the persistence of CD19+ leukemic cells have been linked to the inability of CAR-T cells to overcome the suppressive barrier imposed by Tregs (40).

## 5 Regulatory T cells in Chronic Myeloid Leukemia

CML is a myeloproliferative neoplasm driven by the BCR-ABL1 translocation. While it originates in the BM stem cell compartment, it manifests with massive PB involvement. In the chronic phase (CP) of treatment-naive CML, the systemic immune response becomes gradually exhausted. The PB serves as the primary compartment where robust Treg expansion is observed, acting as a major mechanism of immune evasion prior to the initiation of tyrosine kinase inhibitor (TKI) therapies (41).

CML provides a unique model to study the interaction between a defined oncogene (BCR-ABL1) and the immune system. Treatment-naive CML patients at diagnosis exhibit significantly elevated Treg frequencies compared to healthy controls (42). The BCR-ABL1 kinase activity drives the upregulation of immunomodulatory molecules, including PD-L1 and soluble CD25, which promote Treg expansion (42). A novel mechanism identified in CML involves leukemic exosomes. CML-derived extracellular vesicles (EVs) carrying TGF-beta and other signaling molecules are taken up by T cells, inducing FoxP3 expression and converting them into Tregs (43). This represents a remote-control mechanism by which the leukemia reshapes the immune system systemically, not just in the marrow.

The introduction of TKIs (Imatinib, Dasatinib, Nilotinib) revolutionized CML treatment. Interestingly, these drugs exert off-target effects on the immune system that contribute to their efficacy. For instance, in patients receiving TKI therapy, imatinib has been shown to reduce peripheral Treg frequencies and impair their suppressive function by inhibiting LCK, a kinase downstream of the T-cell receptor (31, 44). This reduction in Treg burden correlates with molecular responses. Dasatinib is a potent inhibitor of SRC family kinases and has a profound immunostimulatory effect. It promotes the expansion of Large Granular Lymphocytes (LGLs), which are cytotoxic NK/T cells, and simultaneously reduces Treg numbers in the PB (45). This immune modulation is thought to contribute to the deep molecular responses (DMR) seen with Dasatinib, which are often deeper and faster than those achieved with Imatinib. The mechanism involves the specific inhibition of LCK in Tregs, which are more sensitive to this inhibition than effector T cells, leading to a selective depletion of the suppressive population (46).

A critical goal in modern CML therapy is Treatment-Free Remission (TFR)—the ability to stop TKI therapy without relapse. The immune system plays a decisive role here. Patients who successfully maintain TFR (molecularly undetectable disease after stopping drugs) tend to have a distinct peripheral immune profile characterized by low Treg numbers and high NK cell function (47). Conversely, early relapse after discontinuation is associated with high Treg/effector ratios. This suggests that in the absence of TKI pressure, the immune system must actively suppress the residual leukemic clone, and Tregs act as a brake on this surveillance. High PB Treg levels at the time of discontinuation are a robust predictor of molecular relapse, suggesting that immune monitoring should be integral to TFR protocols (48).

## 6 Regulatory T cells in Chronic Lymphocytic Leukemia

CLL is uniquely characterized by its profound reliance on secondary lymphoid tissues (lymph nodes and spleen), where characteristic proliferation centers or pseudofollicles reside. While CLL cells circulate abundantly in the PB and infiltrate the BM, the lymph node (LN) microenvironment is the critical sanctuary. Here, Tregs are preferentially recruited via specific chemokine axes (such as CCR7/CCL19/CCL21) and interact directly with CLL cells and nurse-like cells to create a highly immunosuppressive and pro-survival niche (49, 50).

CLL is characterized by profound immunological dysregulation, manifesting paradoxically as both immunodeficiency and autoimmunity. Tregs are significantly expanded in the PB, BM, and LN compartments of both treatment-naive and relapsed CLL patients, both in absolute numbers and percentages (51). These Tregs often exhibit an effector-memory phenotype and express high levels of inhibitory receptors like CTLA-4, PD-1, and TIGIT (52). The expansion of Tregs in the PB and secondary lymphoid organs in CLL correlates with advanced disease stage and poor prognostic markers like ZAP-70 expression (53). The accumulation of Tregs is driven by the CLL cells themselves, which act as ineffective APCs within the LN niches, inducing Treg differentiation rather than effector T cell activation (48).

The PI3K-delta signaling pathway is hyperactive in CLL cells and is critical for their survival. Interestingly, this pathway is also essential for Treg function. The use of PI3K inhibitors like Idelalisib and Duvelisib in relapsed/refractory CLL has revealed a dual mechanism of action: direct cytotoxicity against the leukemic B cells and the depletion of Tregs across tissue compartments (54). However, the depletion of Tregs by Idelalisib can lead to severe autoimmune toxicities (e.g., colitis, hepatotoxicity) because the brakes on the immune system are removed too abruptly or non-specifically. This highlights the delicate balance required when targeting Tregs (55).

Ibrutinib, the first-in-class BTK inhibitor, also affects T cells via inhibition of ITK (Interleukin-2-inducible T-cell Kinase). In the post-treatment setting, this leads to a Th1 skewing of the T cell compartment and a reduction in the Treg/CD4 ratio, which may enhance anti-tumor immunity (56). Newer, more specific BTK inhibitors like Acalabrutinib have less impact on ITK and consequently less effect on T cell populations, which may explain their different toxicity and efficacy profiles (57). Ibrutinib’s ability to reduce systemic and tissue-resident Treg frequency while preserving Th1 immunity contributes to its therapeutic success, although restoration of full immune surveillance remains incomplete (58).

## 7 Prognostic and predictive value of Tregs in leukemia

Before interpreting the broad prognostic trends of Tregs, it is imperative to address how methodological discrepancies (specifically flow cytometry gating strategies, subset definitions, and the biological compartment examined) profoundly shape clinical conclusions. The discordant prognostic findings discussed in this review can largely be reconciled through this methodological lens.

Defining bona fide Tregs in the highly inflammatory leukemic milieu is challenging. Activated effector T cells can transiently upregulate both CD25 and FoxP3. Consequently, in AML, early paradoxical reports suggesting a neutral or even favorable role for Tregs often relied on basic CD4+CD25+ or CD4+FoxP3+ gating. This approach inadvertently captured mixed populations, including activated anti-tumor effector T cells, thereby masking true regulatory suppression. When modern studies employ stringent gating for stable Tregs (incorporating CD127low) or specifically isolate highly suppressive activated effector-Treg subsets (e.g., CD45RA−FoxP3hi), the consensus overwhelmingly shifts. As discussed in Section 3, when studies specifically evaluate subsets with heightened suppressive capacity—such as TNFR2+ (8), PD-1+ (32), or CD39+ (17) Tregs—the correlation with shorter overall survival and chemoresistance becomes remarkably consistent.

The spatial compartmentalization of Tregs significantly limits cross-study comparability. A critical example is in ALL, where the efficacy of T-cell engaging therapies is heavily influenced by Tregs. As highlighted in Section 4, Duell et al. elegantly established a strict peripheral blood (PB) cutoff (>8.525%) for predicting blinatumomab failure (6). However, the subsequent challenge in applying this specific threshold across all pooled trials likely stems from compartmental divergence (38). Circulating Tregs in the PB do not consistently mirror the density, activation state, or suppressive architecture of Tregs residing deep within the bone marrow (BM) niche.

Similarly, the divergent prognostic impact observed between pediatric and adult ALL must be interpreted with caution (36). While biological differences between pediatric and adult hosts play a major role, pediatric studies frequently analyze BM aspirates at diagnosis, whereas many adult correlative studies rely on PB samples. Bone marrow-infiltrating Tregs often exhibit an effector-memory phenotype and are structurally integrated with LSCs, making them biologically distinct from their circulating counterparts (3, 11). Therefore, future clinical trials must prioritize high-dimensional immunophenotyping (e.g., incorporating CD127low, CD45RA, and functional markers like CD39/TIGIT) and rigorously distinguish between PB and BM compartments to establish reliable, reproducible Treg biomarkers.

The clinical significance of Treg infiltration is highly context-dependent, varying significantly across leukemia subtypes, disease stages, and therapeutic interventions. Notably, a clear distinction must be made between the prognostic value of Tregs (reflecting the natural course of the disease and survival outcomes) and their predictive value (indicating the likelihood of response to specific treatments, such as targeted immunotherapies or treatment discontinuation). Furthermore, discrepant findings in the literature often stem from methodological variations, including differences in sample sources (e.g., bone marrow vs. peripheral blood), diverse phenotypic definitions of Tregs (e.g., total CD4+CD25+FoxP3+ cells versus specific hyper-suppressive subsets like TNFR2+ or PD-1+ Tregs), and the exact clinical endpoints evaluated. **Table 1** summarizes the prevailing trends observed in clinical studies.

**Table 1:** Prognostic and predictive value of Tregs by leukemia subtype and clinical context.

Leukemia subtype	Association type	Disease setting	Sample source	Treg definition/subsets	Clinical endpoint	Summary of findings	Ref.
AML	Prognostic	Newly diagnosed/Relapsed	BM and PB	Total CD4+CD25+FoxP3+; Specific subsets: TNFR2+, PD-1+, LAG-3+	OS, DFS	High Treg frequencies generally correlate with poor OS and DFS. Specific subsets (e.g., TNFR2+ or PD-1+ Tregs) and Treg-related gene signatures are stronger independent predictors of poor outcomes than total Tregs alone.	(8, 31, 32, 59)
Adult ALL	Predictive & Prognostic	Relapsed/Refractory (evaluating BiTEs/CAR-T); Initial diagnosis	PB and BM	CD4+CD25+CD127lowFoxP3+; Co-expressing CD39, TIGIT, TIM-3	ORR, Treatment Failure, RFS	High baseline PB Tregs predict non-response to T-cell engagers (blinatumomab) and higher relapse rates following CD19 CAR-T cell therapy. High expression of CD39/TIGIT/TIM-3 on Tregs strongly favors B-ALL progression.	(6, 38, 39, 60)
Pediatric ALL	Prognostic	Newly diagnosed	BM	CD4+CD25+ FoxP3+	EFS, OS	Less clear or neutral association; unlike in adults, high BM Tregs do not consistently predict adverse outcomes.	(36)
CML	Predictive	Chronic phase (evaluating TKI discontinuation)	PB	CD4+CD25+ FoxP3+	Treatment-Free Remission (TFR), Molecular relapse	Low Treg numbers combined with restored NK cell function are critical prerequisites for TFR. An “effector-suppressor score” integrating low FoxP3+ Tregs and high NK cells effectively predicts successful TFR prior to discontinuation.	(41, 47, 48, 61)
CLL	Prognostic	Early-stage (Watch & Wait); Post-first-line therapy	PB	CD4+CD25high FoxP3+	Time to First Treatment (TTFT), Time to Next Treatment (TTNT), OS	High Treg frequency at diagnosis correlates with advanced Rai stage and significantly shorter TTFT. In treated patients, elevated Tregs predict shorter TTNT and reduced overall survival.	(4, 53, 62)

## 8 Tregs in Hematopoietic Stem Cell Transplantation

To fully grasp the clinical implications of Tregs in hematologic malignancies, it is crucial to clearly distinguish between their role in primary leukemia biology and their function in allo-HSCT-related immunoregulation. In the endogenous leukemic microenvironment, host-derived Tregs are co-opted by the malignancy to suppress immunosurveillance, serving as a shield that promotes immune evasion and tumor progression. In this context, as discussed in previous sections, therapeutic strategies aim to deplete or inhibit Tregs to restore anti-leukemic immunity. Conversely, in the allo-HSCT setting, the immunological landscape and the primary origin of the Tregs (which are typically donor-derived) shift dramatically. The predominant clinical threat is no longer solely tumor-driven immune suppression, but rather the overwhelming, potentially lethal alloreactivity of donor immune cells against recipient tissues. Here, donor Tregs are highly desirable because they act as essential brakes on the donor effector T cells responsible for Graft-versus-Host Disease (GvHD) (63).

Allogeneic HSCT remains the only curative option for many high-risk leukemias. The central paradox of this therapy is that its efficacy relies on the Graft-versus-Leukemia (GvL) effect, where alloreactive donor T cells eradicate residual host leukemic cells, which is inextricably linked to the detrimental GvHD, where these same donor T cells attack healthy host epithelial tissues (64). High numbers of Tregs in the donor graft or rapid Treg reconstitution post-transplant are strongly associated with a reduced incidence of acute and chronic GvHD (65). The historical concern has been that too many donor Tregs would suppress the beneficial GvL effect, leading to relapse. However, recent data challenges this dogma. Clinical data suggests that donor Tregs may preferentially suppress the intense inflammation of GvHD without completely abrogating the GvL effect (63). This separation of function is hypothesized to occur because Tregs more effectively control broad tissue inflammation rather than discrete, antigen-driven cytotoxic responses against hematopoietic targets (64). Achieving this perfect separation of function remains the holy grail of transplant immunology.

Given their indispensable protective role against GvHD, therapeutic strategies in the HSCT setting are diametrically opposed to those in non-transplant settings: instead of depleting Tregs, the goal is to enhance them. There is immense interest in the adoptive transfer of ex vivo expanded donor-derived Tregs. Clinical trials (e.g., the TRACT trial) have infused polyclonal Tregs into transplant recipients. Results have shown safety and a reduction in GvHD, with no apparent increase in leukemia relapse rates (66). However, the specificity of polyclonal Tregs is low, carrying a residual risk of generalized immunosuppression that might eventually blunt the GvL effect. Future strategies involve CAR-Tregs (e.g., HLA-A2 specific CAR-Tregs) that can home specifically to the transplant interface to provide localized immunosuppression. Preclinical models show that these A2-CAR Tregs can prevent rejection and GvHD more potently than polyclonal Tregs, theoretically sparing systemic anti-tumor immunity by localizing suppression to the site of antigen mismatch (67). More recently, innovative strategies such as CD19-directed CAR-Tregs have been explored. In murine models of allo-HSCT, these target-specific CAR-Tregs effectively suppressed lethal acute GvHD while unexpectedly maintaining robust GvT (Graft-versus-Tumor) responses against malignant B cells, proving that highly engineered Tregs can uncouple GvHD toxicity from therapeutic GvL efficacy (68).

## 9 Therapeutic targeting of Tregs

**Figure 3:**
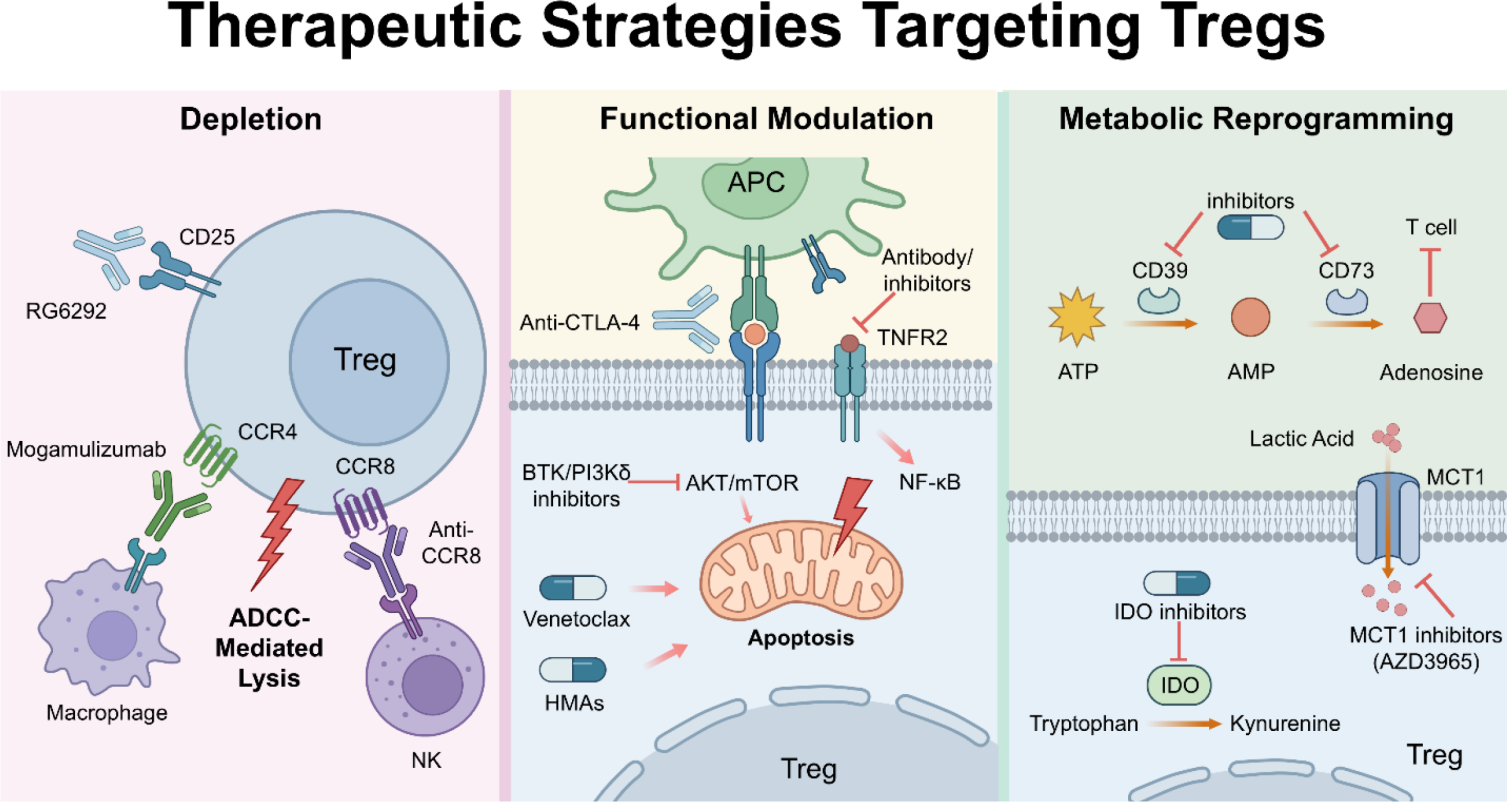
Therapeutic strategies targeting Tregs: depletion, functional modulation, and metabolic reprogramming.

To restore anti-leukemic immunity, multiple strategies are being developed to deplete Tregs, inhibit their function, or destabilize their lineage (**Figure 3**).

### 9.1 Monoclonal antibodies for depletion

#### 9.1.1 Anti-CD25 (Daclizumab, Basiliximab, RG6292)

CD25 is a canonical Treg marker, yet its utility as a target is complicated by its expression on activated effector T cells. First-generation antibodies (e.g., Daclizumab, Basiliximab) block IL-2 binding. While they deplete Tregs, they also starve effector T cells of IL-2, which can impair the anti-tumor response (69).

Novel antibodies like RG6292 (vopikitug) are designed to deplete Tregs via Antibody-Dependent Cellular Cytotoxicity (ADCC) without blocking IL-2 signaling to effector cells (70). This allows for Treg elimination while preserving the “good” T cells ability to proliferate in response to IL-2. Clinical trials combining RG6292 with atezolizumab (anti-PD-L1) have shown dose-dependent Treg depletion and manageable safety profiles, although efficacy as a monotherapy in solid tumors has been modest, prompting investigation into leukemia where Treg burden is higher (71). Furthermore, bispecific antibodies (e.g., CD25xTIGIT) have shown promise in preclinical models by selectively depleting intratumoral Tregs (which express both markers) while sparing peripheral T cells. This dual targeting minimizes “on-target, off-tumor” toxicity (72).

#### 9.1.2 Anti-CCR4 (Mogamulizumab)

Mogamulizumab targets CCR4, the chemokine receptor used by Tregs to traffic to the tumor and it can deplete CCR4+ Tregs via ADCC. In AML and solid tumors, it effectively reduces Treg numbers. However, its use is complicated by severe skin toxicities because CCR4 is also a homing receptor for skin-resident T cells (10). Trials combining Mogamulizumab with PD-1 inhibitors are ongoing to see if Treg depletion sensitizes tumors to checkpoint blockade (73).

#### 9.1.3 Anti-CCR8

CCR8 is emerging as a highly specific marker for tumor-infiltrating Tregs, with low expression on peripheral Tregs or effector cells. Anti-CCR8 antibodies (e.g., DT-7012) are showing potent efficacy in depleting the most suppressive Treg subsets within the tumor bed with less systemic toxicity than anti-CD25 or anti-CCR4 (74). The specificity of CCR8 for intratumoral Tregs makes it an ideal target for surgical immune depletion, sparing systemic tolerance (75).

### 9.2 Metabolic modulation

#### 9.2.1 IDO inhibitors (Epacadostat)

Inhibiting IDO should theoretically prevent Treg induction. While Epacadostat failed in phase III melanoma trials (ECHO-301), interest remains in leukemia (76).The failure in solid tumors may have been due to compensatory pathways (e.g., TDO upregulation). In AML, where IDO expression is high on blasts, combinations with chemotherapy or hypomethylating agents are still being explored to disrupt the kynurenine-driven Treg induction loop (77).

#### 9.2.2 Adenosine pathway (CD39/CD73) blockade

Antibodies targeting CD39 or CD73 inhibit the production of immunosuppressive adenosine. This relieves the metabolic brake on effector T cells and prevents the stabilization of Tregs. This strategy is particularly relevant in AML, where the adenosine pathway is upregulated and contributes significantly to immune evasion (16). Combined blockade of CD73 and other checkpoints like TIGIT has shown synergistic effects in preclinical models, restoring NK and T cell cytotoxicity (78).

### 9.3 Small molecule drugs

#### 9.3.1 Cyclophosphamide (low-dose)

Metronomic low-dose cyclophosphamide selectively depletes Tregs because they have lower intracellular ATP levels and reduced glutathione capacity compared to effector cells, making them more sensitive to alkylating agents (79). This “old drug, new trick” approach is being used to prime patients for vaccines or immunotherapy (80).

#### 9.3.2 Venetoclax

The BCL-2 inhibitor Venetoclax, a standard of care in AML, has interesting immune effects. It increases Reactive Oxygen Species (ROS) generation in T cells, which enhances the effector function of cytotoxic T cells (81). It sensitizes AML cells to T-cell killing and does not appear to impair T cell function, unlike cytotoxic chemotherapy (82). In fact, Venetoclax has been shown to reduce the frequency of cytokine-secreting non-suppressive Tregs and modulate immune checkpoints like TIM-3, creating a more favorable immune profile (83).

#### 9.3.3 Hypomethylating Agents

Azacitidine and Decitabine induce the expression of “viral mimicry” genes (endogenous retroviruses) in leukemic cells, causing an interferon response that attracts T cells. They also appear to directly reduce FoxP3 expression in Tregs, destabilizing their lineage (84). Although some studies suggest this might transiently increase FoxP3 expression but result in non-suppressive and unstable Tregs, the net clinical effect is often a sensitization to immune attack (85). .

Current approaches include three main modalities: (1) Depletion: Monoclonal antibodies (e.g., anti-CD25, anti-CCR4, anti-CCR8) eliminate highly suppressive Tregs via macrophage and NK cell-mediated ADCC. (2) Functional Modulation: Immune checkpoint blockades (e.g., anti-CTLA-4, anti-TNFR2) and small molecules (e.g., venetoclax, HMAs) disrupt Treg suppressive capacity and intracellular signaling, promoting apoptosis. (3) Metabolic Reprogramming: Inhibitors targeting the CD39/CD73 axis, IDO pathway, or MCT1 alleviate immune suppression by preventing the generation of immunosuppressive metabolites (e.g., adenosine, kynurenine) and depriving Tregs of metabolic substrates.

## 10 The challenge of Tregs in CAR-T cell therapy

Tregs represent a major, underappreciated mechanism of resistance to CAR-T therapy in leukemia. When CAR-T cells are infused, the host’s Tregs can suppress them via: (1) IL-10 Production: Blunting the CAR-T cytotoxic response (6); (2) Competition for IL-2: Tregs act as IL-2 sinks, starving the CAR-T cells and preventing their proliferation (86); (3) Inhibitory Checkpoints: Upregulation of PD-L1/LAG-3 on Tregs interacting with CARs (87).

**Figure 4:**
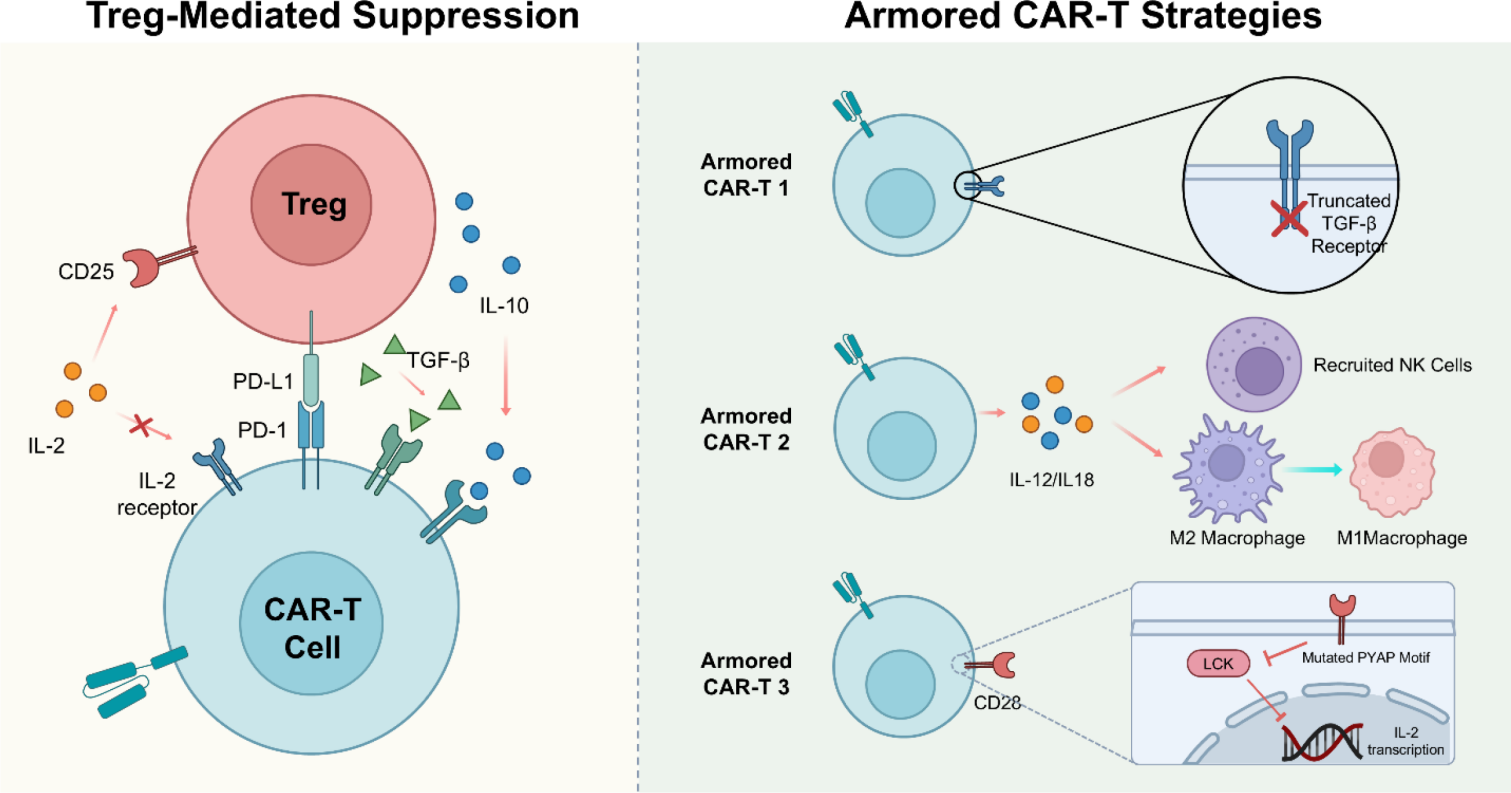
Treg-mediated suppression of CAR-T cells and armored CAR-T engineering strategies.

To circumvent these barriers, next-generation CAR designs incorporate specific countermeasures (**Figure 4**). CAR-T cells expressing a truncated TGF-β receptor can bind the cytokine (removing it from the environment) but do not transmit the inhibitory signal. This renders them impervious to Treg-derived TGF-β (88). Preclinical studies in solid tumors and hematologic models have shown that these armored CARs persist longer and eradicate tumors even in TGF-β-rich environments (89). CAR-T cells engineered to secrete IL-12 can repolarize the local TME, converting Tregs into Th1-like cells or negating their suppression (90). This autocrine cytokine support makes the CAR-T cells resistant to Treg-mediated inhibition and enhances their cytotoxicity against leukemic blasts. Altering the Lck binding domain of the CAR costimulatory region to reduce IL-2 production (which feeds Tregs) while maintaining CAR proliferation is another innovative strategy (86). This starves the local Tregs while allowing the CAR-T cells to function.

Despite the promising potential of armored CAR-T cells to overcome Treg-mediated suppression, these engineering strategies introduce significant safety concerns that must be carefully managed. The constitutive secretion of pro-inflammatory cytokines, such as IL-12, while highly effective at counteracting the suppressive TME, poses a severe risk of systemic toxicities, including life-threatening cytokine release syndrome (CRS) and immune effector cell-associated neurotoxicity syndrome (ICANS) (91). Similarly, strategies that completely abrogate inhibitory signaling, such as the expression of dominant-negative TGF-β receptors (dnTGF-βRII), risk breaking peripheral immune tolerance. Because TGF-β signaling is critical for maintaining normal tissue homeostasis and preventing autoimmunity, rendering CAR-T cells universally insensitive to TGF-β could lead to uncontrolled T-cell proliferation and severe autoimmune-like pathologies (92).

To mitigate these risks and achieve a sustainable therapeutic window, contemporary armored CAR-T designs increasingly incorporate sophisticated genetic control mechanisms. One prominent approach is the integration of suicide switches, such as the inducible caspase-9 (iCasp9) or herpes simplex virus thymidine kinase (HSV-TK) systems. The iCasp9 system, for instance, allows for the rapid and selective induction of apoptosis in hyperactive CAR-T cells upon the administration of a small-molecule dimerizer (e.g., AP1903), providing a reliable fail-safe in the event of severe toxicity (93, 94). Alternatively, to prevent systemic toxicity from the outset, researchers are employing inducible expression systems to restrict cytokine release strictly to the tumor site. By utilizing T-cell activation-dependent promoters (such as NFAT-responsive elements) or synthetic Notch (synNotch) receptors, CAR-T cells are engineered to secrete IL-12 or other potent payloads exclusively upon engaging specific tumor antigens (95, 96). These logic-gated and locally restricted strategies ensure that the anti-leukemic functions of armored CAR-T cells are confined to the targeted microenvironment, maximizing efficacy while preserving systemic safety. .

Treg-Mediated Suppression: Tregs hinder CAR-T cell efficacy through the secretion of inhibitory cytokines (IL-10, TGF-β), upregulation of immune checkpoints (PD-L1), and competitive deprivation of IL-2. Armored CAR-T Strategies: Next-generation CAR-T cells overcome these barriers through specific modifications, including the expression of dominant-negative TGF-β receptors to resist suppression (Armored CAR-T 1), constitutive secretion of pro-inflammatory cytokines (IL-12/IL-18) to remodel the local immune niche (Armored CAR-T 2), and CD28 costimulatory domain mutations (PYAP motif) to starve surrounding Tregs of CAR-T-derived IL-2 (Armored CAR-T 3).

## 11 Conclusion and future perspectives

The exploration of Regulatory T cells in leukemia has evolved from a simplistic view of them as mere suppressors of anti-tumor immunity to a nuanced understanding of their role as central architects of the leukemic bone marrow niche. As synthesized in this review, leukemic blasts actively co-opt Treg biology through complex chemokine networks (e.g., CCL22/CCR4), metabolic symbiosis (CD39/CD73 axis, IDO metabolism), and direct cell-contact mechanisms to construct an immune sanctuary that favors leukemic stem cell persistence and therapeutic resistance. While the prognostic impact of Tregs varies across leukemia subtypes—largely deleterious in AML and adult ALL, yet context-dependent in pediatric cases and post-transplant settings—their pivotal role in hindering the efficacy of modern immunotherapies, including BiTEs and CAR-T cells, is indisputable.

Despite the significant progress in delineating these mechanisms, translating this knowledge into curative clinical strategies requires navigating several critical challenges. Foremost among these is the shift from broad suppression to precision targeting. The historical strategy of systemic Treg depletion (e.g., via anti-CD25) has been hampered by the inadvertent elimination of activated effector T cells and the risk of severe autoimmunity. Consequently, the future of Treg-targeted therapy lies in discerning and targeting the specific subsets responsible for intratumoral suppression. Emerging markers such as CCR8 and TNFR2 offer a promising avenue to selectively ablate highly suppressive, tumor-infiltrating Tregs while preserving the peripheral regulatory pool required for systemic tolerance. Clinical validation of anti-CCR8 antibodies and bispecific constructs (e.g., TIGIT/CD25) in hematologic malignancies will be a priority in the coming years.

Beyond depletion, a paradigm shift is emerging toward reprogramming Tregs, capitalizing on the plasticity of T cells within the hypoxic and lactate-rich leukemic niche. Targeting the metabolic vulnerabilities of leukemic Tregs—specifically their OXPHOS dependency and the adenosine-generating CD39/CD73 pathway—represents a strategy to destabilize their lineage rather than simply killing them. Theoretically, combining metabolic modulators (such as IDO inhibitors or adenosine receptor antagonists) with checkpoint blockade could convert the immunosuppressive TME into an immunostimulatory one, potentially restoring the cytotoxic function of exhausted T cells without the toxicity associated with broad depletion.

However, realizing the full potential of these strategies necessitates a deeper understanding of the bone marrow’s spatial architecture. Most current data rely on flow cytometry or bulk RNA sequencing, which dissociates cells from their native context. The application of spatial transcriptomics and high-dimensional single-cell analysis to bone marrow trephines will be crucial to visualize the neighborhoods where Tregs interact with LSCs. Understanding the specific spatial cues that anchor Tregs in the vascular niche could reveal novel targets to mobilize LSCs from their dormant state, rendering them susceptible to chemotherapy.

Simultaneously, as cellular therapies move to the forefront of leukemia treatment, overcoming the Treg barrier is non-negotiable. The next generation of armored CAR-T cells, engineered with dominant-negative TGF-β receptors or autocrine cytokine loops (e.g., IL-12, IL-18), represents a definitive step toward rendering effectors impervious to the suppressive TME. Furthermore, investigating the timing of Treg modulation—perhaps as a conditioning strategy prior to T-cell apheresis or infusion—may optimize the fitness of the final cell product.

In conclusion, the therapeutic manipulation of Tregs in leukemia is moving toward a precision medicine era. The ultimate goal is not merely to remove the brakes of the immune system, but to intricately remodel the bone marrow microenvironment, decoupling the graft-versus-leukemia effect from graft-versus-host disease, and restoring durable immunosurveillance against malignant clones.
